# Case report: A fatal case of myocardial infarction due to myocardial bridge and concomitant vasospasm: the role of stress gated SPECT

**DOI:** 10.3389/fcvm.2023.1188095

**Published:** 2023-05-30

**Authors:** Seok Oh, Dae Young Hyun, Sang-Geon Cho, Young Joon Hong, Ju Han Kim, Youngkeun Ahn, Myung Ho Jeong

**Affiliations:** ^1^ Department of Cardiology, Department of Internal Medicine, Chonnam National University Hospital, Gwangju, Republic of Korea; ^2^ Department of Nuclear Medicine, Chonnam National University Hospital, Gwangju, Republic of Korea; ^3^ Department of Cardiology, Department of Internal Medicine, Chonnam National University Medical School, Gwangju, Republic of Korea

**Keywords:** cardiac imaging techniques, myocardial bridging, myocardial infarction, coronary angiography, SPECT CT

## Abstract

**Introduction:**

Although most cases of myocardial bridge (MB) are clinically benign, sometimes it can be one of potential threats of myocardial infarction (MI) and life-threatening arrhythmia. In the present study, we present a case of ST-segment elevation MI caused by MB and concomitant vasospasm.

**Case Presentation:**

A 52-year-old woman was brought to our tertiary hospital due to resuscitated cardiac arrest. Because the 12-lead electrocardiogram indicated ST-segment elevation MI, coronary angiogram was promptly commenced, which showed near-total occlusion at the middle portion of left anterior descending coronary artery (LAD). After intracoronary nitroglycerin administration, this occlusion was dramatically relieved, however, systolic compression at this site remained, indicative of myocardial bridge (MB). Intravascular ultrasound also showed eccentric compression with a “half-moon” sign, which is consistent with MB. Coronary computed tomography also showed a bridged coronary segment surrounded by myocardium at the middle portion of LAD. To assess the severity and extent of myocardial damages and ischemia, myocardial single photon emission computed tomography (SPECT) was additionally conducted, showing a moderate fixed perfusion defect around the cardiac apex, suggesting MI. After receiving optimal medical therapy, the patient's clinical symptoms and signs were improved then the patient was discharged from the hospital successfully and uneventfully.

**Conclusion:**

We demonstrated a case of MB-induced ST-segment elevation MI which was confirmed with its perfusion defects via myocardial perfusion SPECT. There have been proposed a number of diagnostic modalities to examine its anatomic and physiologic significance. Among them, myocardial perfusion SPECT can be available as one of useful modalities to evaluate the severity and extent of myocardial ischemia in patients with MB.

## 1. Introduction

Myocardial bridge (MB) is a congenital coronary anomaly manifested by coronary artery segment tunnelling through the myocardial bands (1), and this term was first mentioned in 1961 in a case report about angiographic narrowing during the systole (2). Although many clinicians tend to consider it to be a benign condition, sometimes it can be clinically fatal (3, 4). In other words, MB may contribute to the development of acute coronary syndrome such as myocardial infarction (MI) or cardiac arrest (5–7). Especially, coronary vasospasm (CVS) may act as a trigger factor for these situations in patients with MB (7). We present a case of ST-segment elevation MI triggered by CVS within the site of MB.

## 2. Case presentation

**Figure 1:**
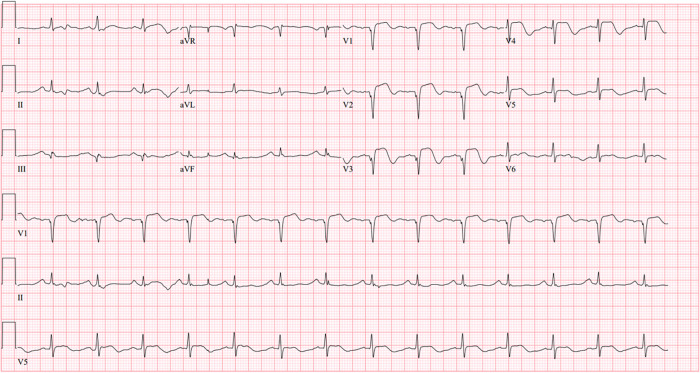
Initial 12-lead electrocardiogram: A 12-lead electrocardiogram showed abnormal ST-segment elevation with pathologic Q-waves in precordial leads.

A 52-year-old Korean woman with essential hypertension was admitted to our cardiovascular center because of resuscitated cardiac arrest as the chief complaint. Several hours before the presentation, the patient experienced squeezing chest pain and was suddenly collapsed then received bystander cardiopulmonary resuscitation. During the transportation via ambulance, the patient had received electrical defibrillation for documented ventricular fibrillation (Supplementary Figure S1). There was no documentation of previous cardiovascular events. On the physical examination, temperature was 36.5°C, heart rate was 90 beats per minute, respiratory rate was 20 beats per minute, and blood pressure was 120/90 mmHg. A 12-lead electrocardiogram showed abnormal ST-segment elevation with pathologic Q-waves in precordial leads (Figure 1). In the laboratory test, high-sensitivity troponin-I was elevated to 1.332 ng/ml (reference: 0–0.050 ng/ml). Since we initially concluded a diagnosis of ST-segment elevation MI, the catheterization laboratory was activated then emergent coronary angiogram (CAG) was promptly planned for percutaneous coronary intervention.

**Figure 2:**
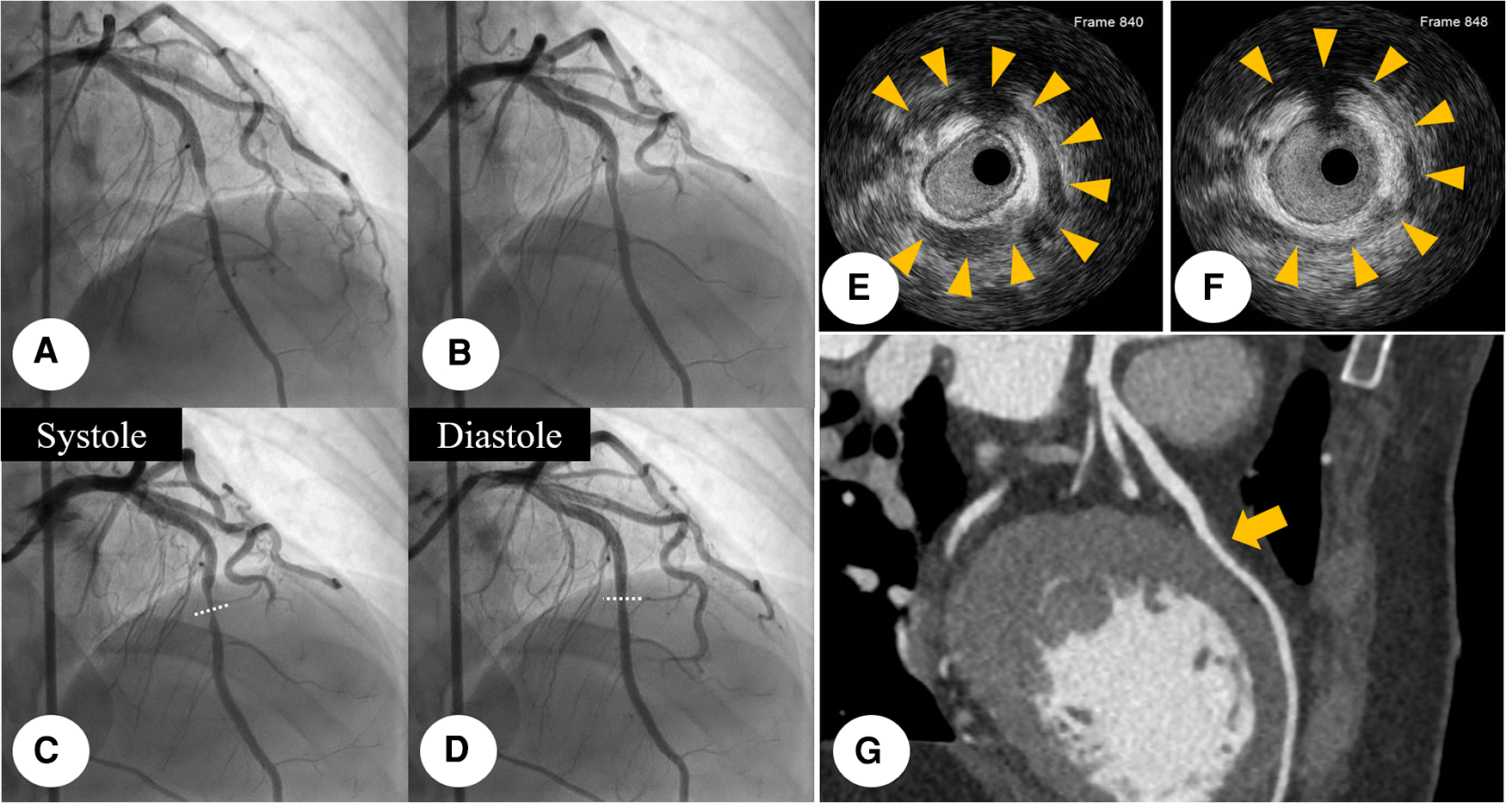
Initial CAG finding: initial CAG demonstrated near-total occlusion (yellowish arrow) at the middle portion of LAD with reduced antegrade coronary flow. (**A**) This stenosis was dramatically relieved (yellowish arrow), after I-NTG administration (**B**) After I-NTG administration, systolic compression was seen at the middle portion of LAD, which means MB. (**C,D**) IVUS was further examined with a guidance system (Eagle Eye® Platinum RX Digital IVUS Catheter, Volcano Corporation, Rancho Cordova, CA, USA), demonstrating eccentric compression with a half-moon-like echo-lucent space between the MB segment and epicardial tissue. (**E,F**) Multiple-slice CCTA imaging showed a bridged coronary segment surrounded by myocardium at the middle portion of LAD (yellowish arrow). (**G**) CAG, coronary angiogram; CCTA, coronary computed tomography angiography; I-NTG, intracoronary nitroglycerin; LAD, left anterior descending coronary artery; MB, myocardial bridge.

Initial CAG was performed via the right femoral artery at the catheterization laboratory. It revealed near-total occlusion at the middle portion of left anterior descending coronary artery (LAD) with reduced antegrade coronary flow (Figure 2A). After intracoronary nitroglycerin (I-NTG) administration, however, this stenosis was dramatically relieved (Figure 2B), but there was found systolic compression at the same site, suggestive of MB (Figures 2C,D, Supplementary Video S1). In quantitative coronary analysis, the systolic diameter was 0.84 mm and the diastolic diameter was 1.17 mm, which meant that the diameter change from diastole to systole was about 28.2% (Supplementary Figure S2). For the further evaluation, intravascular ultrasound (IVUS) study was examined with a guidance system (Eagle Eye® Platinum RX Digital IVUS Catheter, Volcano Corporation, Rancho Cordova, CA, USA). In IVUS, eccentric compression was seen in the MB segment with a half-moon-like echo-lucent space between the MB segment and epicardial tissue (Figures 2E,F, Supplementary Video S2). There was no definite evidence of atherosclerotic plaque formation within the MB segment. The cross-sectional area at this segment was 4.99 mm^2^ during the diastole, and 3.42 mm^2^ during the systole (Supplementary Figure S3). In multiple-slice coronary computed tomography angiography (CCTA), there was seen a coronary segment surrounded by myocardium at the middle portion of LAD (Figure 2G). We concluded that the final diagnosis of this patient was MI with non-obstructive coronary arteries (MINOCA). The patient was transferred to the intensive care unit for the hemodynamic monitoring.

**Figure 3:**
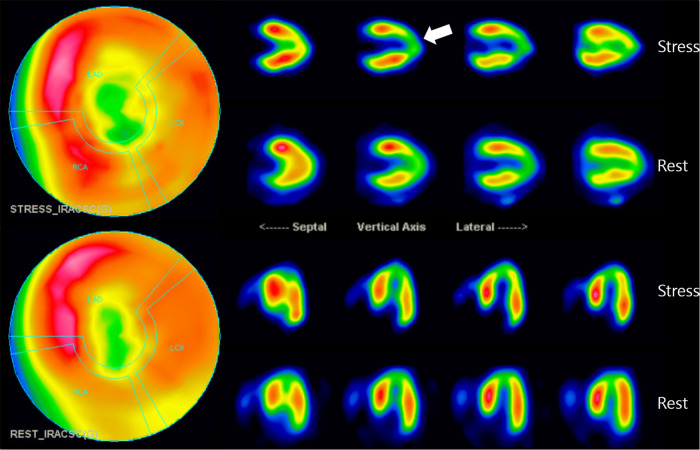
Myocardial perfusion SPECT imaging: SPECT scan showed medium-sized, non-transmural infarction around the apex (whitish arrow). This defect correlates to the downstream myocardium to the bridging segment of the mid-LAD, as marked by arrows. LAD, left anterior descending coronary artery; SPECT, single photon emission computed tomography.

**Figure 4:**
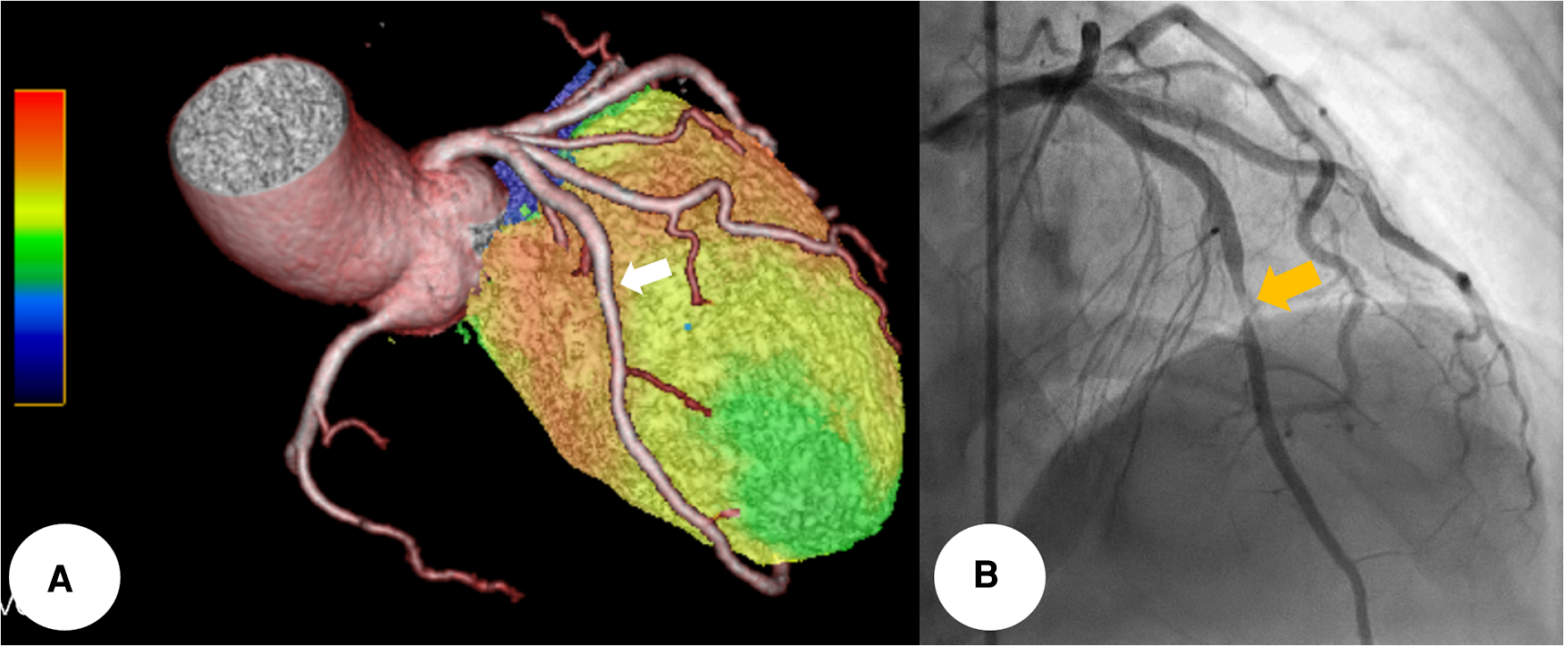
The cardiac SPECT/CCTA hybrid imaging also confirmed excellent correlation between the extent of perfusion defects and the anatomical location of MB, which was well correlated with CAG finding (whitish arrow and yellowish arrow) (**A,B**). CCTA, coronary computed tomography angiography; MB, myocardial bridge; SPECT, single photon emission computed tomography.

To determine its functional significance, Tc-99 m methylisobutyl isonitrile single photon emission computed tomography (SPECT) was additionally conducted. It demonstrated a medium-sized, moderate fixed perfusion defect around the apex, downstream to the bridging segment of the LAD, suggesting MI (Figure 3). The cardiac SPECT/CCTA hybrid imaging also confirmed excellent correlation between the extent of perfusion defects and the anatomical location of MB (Figure 4A), which was also correlated with CAG finding (Figure 4B).

As the patient received optimal antiplatelet agents, high-intensity statins, and non-dihydropyridine calcium channel blockers (CCBs), the patient's clinical symptoms and signs were improved. The patient was discharged from the hospital then has remained symptom-free on follow-up outpatient visits.

## 3. Discussion and conclusion

MB is one of normal variants of coronary artery which is manifested by intramuscular course of a coronary artery. Although the true prevalence of MB is not fully understood, its angiographic detection seems not uncommon with the detection rates varying from 0.5–12% in the resting CAG to about 40% in I-NTG administration or coronary reactivity testing (8). Since most patients with MBs have no clinical symptoms, many clinicians are reluctant to manage it with intensive medical treatments then just tend to recommend simple observation with not much clinical significance. Although most cases with MBs are clinically benign (7), as suggested in the present case, sometimes it can be one of potential threats of MI and life-threatening arrhythmia (9, 10). According to an observational retrospective study, MB seems to be a potential cause of MI, and its prevalence was significantly higher in patients with MINOCA than in their counterparts (10). Moreover, since MB itself is one of independent risk factors for ischemia-induced myocardial fibrosis and MINOCA (11), it also act as a crucial factor contributing to the onset of fatal arrhythmia (12).

There have been proposed a number of diagnostic modalities to examine its anatomic and physiologic significance (13, 14). Invasive modalities include CAG, and intracoronary imaging/physiology studies. CAG may demonstrate the diameter change between systole and diastole within the segment of MB. “Milking effect” is one of characteristic findings with a significant (≥70%) reduction in minimum lumen diameter (MLA) during the systole and persistent ≥35% decrease in MLA (15). After I-NTG administration, systolic compression can be accentuated by vasodilation of non-bridged coronary segments (16). As shown in the present case, IVUS can show the characteristic “half-moon” sign which is an echo-lucent space between the MB segment and epicardial tissue. Atherosclerotic plaque can also be seen proximal to MB in IVUS. Coronary physiologic studies such as fractional flow reserve can be useful, but its role of MB has remained challenging (14).

Non-invasive modalities include CCTA, stress gated SPECT, and stress echocardiogram. CCTA is able to identify bridging segment surrounded by myocardium (17), whereas stress gated SPECT can visualize myocardial perfusion defects then quantify the degree of myocardial ischemia (18). In the present case, we utilized the myocardial perfusion SPECT. Although most cases of MB can be found in anatomical imaging modalities, functional imaging like myocardial perfusion SPECT can also be feasible and useful to evaluate its functional significance in the form of perfusion defects (19). Lee and his colleagues demonstrated that high-grade MB could induce perfusion defects on territories of LAD and its branches in dipyridamole TI-201 SPECT findings (20). Such findings may reflect the degree of luminal narrowing by systolic contraction (18). A retrospective study by Huang et al. showed abnormal myocardial perfusion in patients with MB at the middle portion of LAD (21).

The treatment options can be subdivided into two categories: (A) pharmacological therapy; (B) interventional therapy. In the pharmacological therapy, beta-blocker is the mainstay treatment which can relieve the hemodynamic disturbance caused by MB through its negative chronotropic effect (15). CCBs are also available with similar pharmacologic mechanisms of beta-blockers. Like in this case, they may be more beneficial in patients with MB and concomitant CVS (13). In the literature review, there have been no available comparative studies of beta-blocker vs. CCB in patients with MB. In contrast, pure vasodilators such as long-acting nitrates should be avoided or applied with caution because they can worsen symptoms by accentuating systolic compression of the MB segment (22). If refractory to pharmacological therapy, interventional therapy should be considered. Interventional therapy includes percutaneous coronary intervention with stenting for the bridged coronary segment, supra-arterial myotomy, or coronary artery bypass grafting.

Meanwhile, we should know that this case shows MINOCA with concomitant presence of MB and CVS. MB seems to be closely related to CVS (11, 23, 24). That is, patients having MB tended to have a higher proportion of CVS compared to those not having MB (11, 25). Although not fully accountable, it is plausible that sustained shear stress (i.e., contraction-relaxation effect) of the site of MB may induce the alteration in endothelial function (11, 23), resulting in coronary vasomotor disorders. Since patients with both of them tend to have a higher risk of adverse cardiac events such as readmission for recurrent angina pectoris (11, 25), therefore, coronary reactivity testing may be a useful diagnostic tool to elicit CVS then verify these high-risk patients (26).

In the present case, we detected the fatal case of MB-induced MINOCA through multimodality imaging tools. While there are a number of useful diagnostic modalities, myocardial perfusion SPECT can be available as one of useful modalities to evaluate the severity and extent of myocardial ischemia in patients with MB.

## Data Availability

The original contributions presented in the study are included in the article/**Supplementary Material**, further inquiries can be directed to the corresponding author.
