# Supplementary figures and images for: Case report: A fatal case of myocardial infarction due to myocardial bridge and concomitant vasospasm: the role of stress gated SPECT
Source: Front Cardiovasc Med. 2023 May 30;10:1188095. doi: 10.3389/fcvm.2023.1188095 (PMC10265674; doi:10.3389/fcvm.2023.1188095)

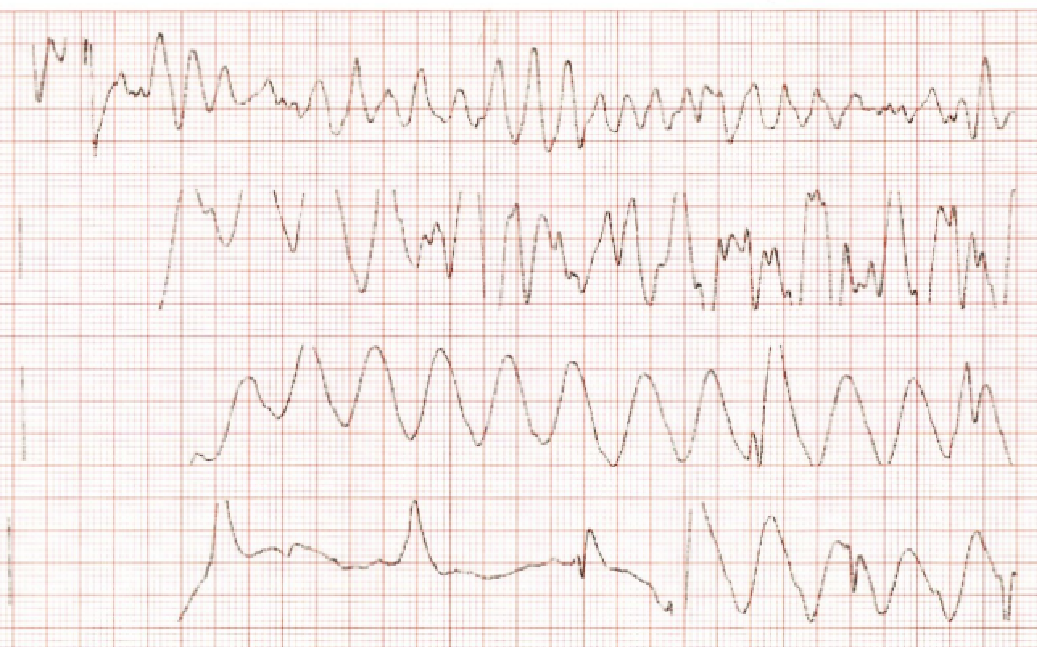

Supplement: Supplementary file 3 [file Image1.tif]

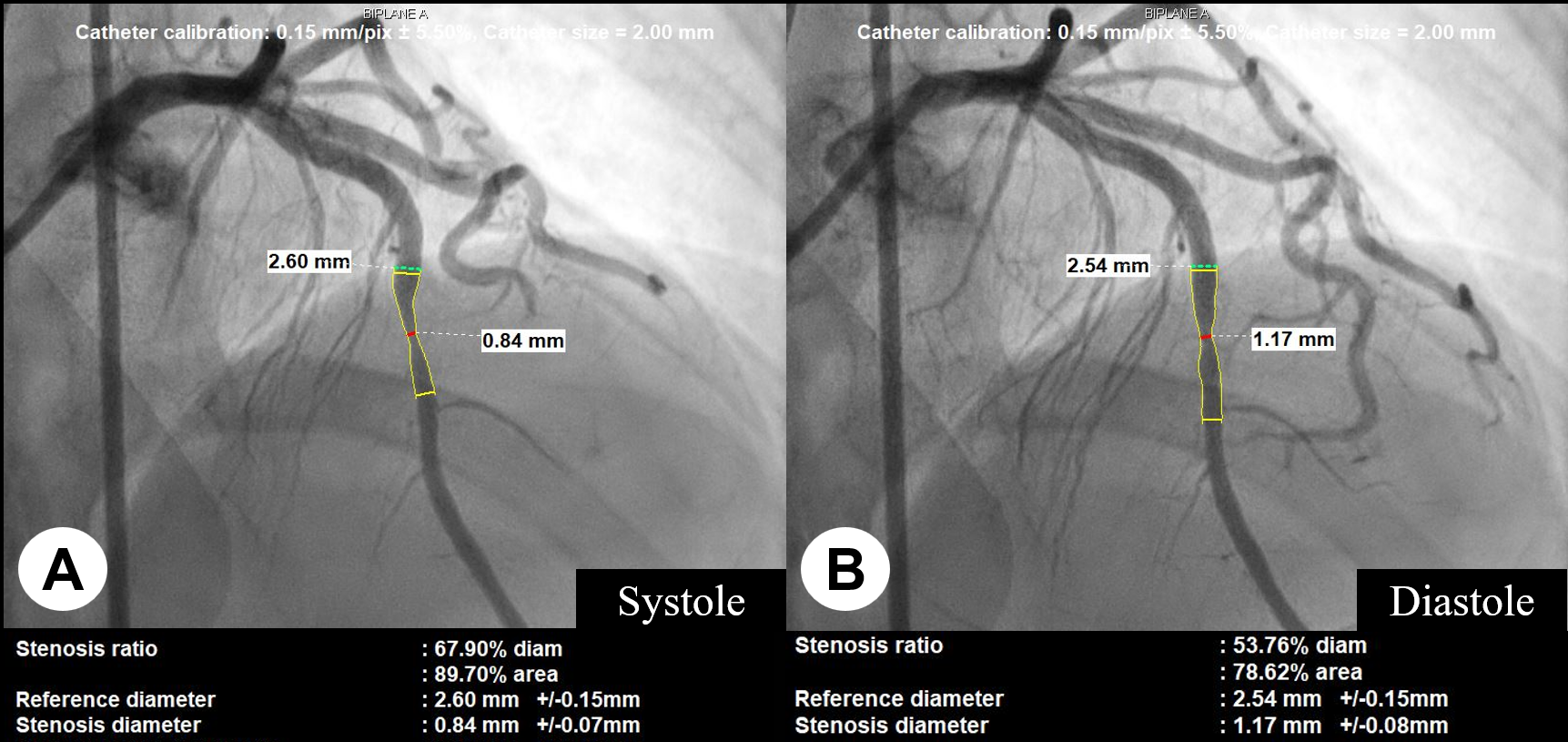

Supplement: Supplementary file 4 [file Image2.tif]

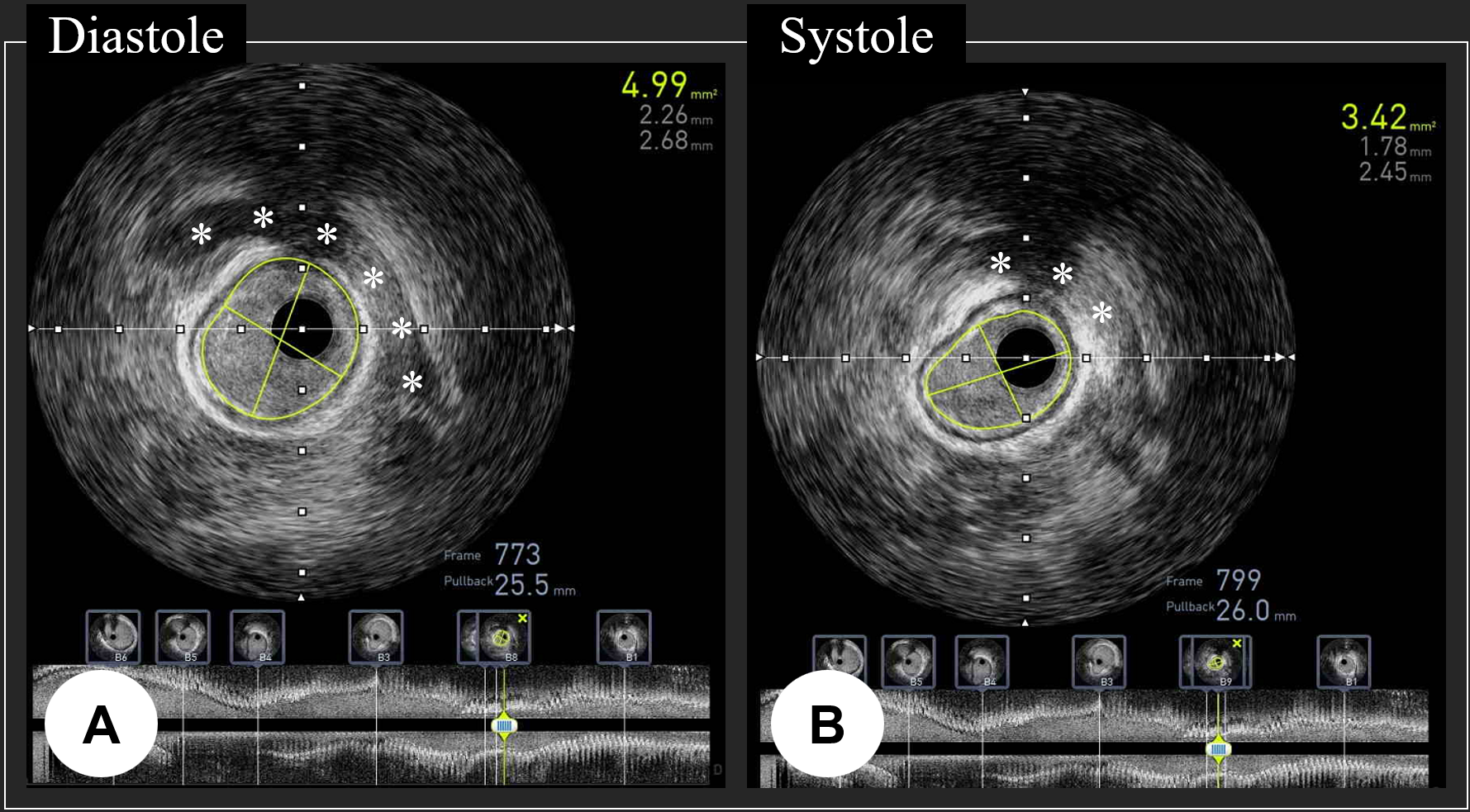

Supplement: Supplementary file 5 [file Image3.tif]
